# Features of Cytokine Profile in Different Age Groups

**DOI:** 10.5195/cajgh.2014.173

**Published:** 2014-12-12

**Authors:** Alikhan Shortanbayev, Beibitgul Bizhigitova, Anel Tarabayeva, Aliya Nurmuchanbetova

**Affiliations:** Department of General Immunology, S.D. Asfendiyarov Kazakh National Medical University, Almaty, Kazakhstan

**Keywords:** aging, cytokines, immunity

## Abstract

**Introduction:**

The study of the cytokine profile during aging is interesting because age-related changes of the immune status are usually correlate with the onset of specific diseases. Characteristics of cytokine activity in the elderly can not only detail the pathogenesis of the disease but also help to choose the appropriate therapeutic strategy, which in addition to the therapeutic effect could improve the quality of life of the elderly. The purpose of this study was to examine cytokine levels in older adults.

**Material and methods:**

We examined 268 people aged 45–80 years and older. All surveyed individuals were divided into 8 different age groups. All participants were tested for concentrations of IL-1β, IL-2, TNF-α and IFN-γ.

**Results:**

The study found that concentrations of TNF-α increased with age. For age group 45–49, the concentration of TNF-α was 5.94 pcg/ml. In older age groups, there was a gradual increase in cytokine concentration. In a group of centenarians, concentration of TNF-α reached 20.55 pcg/ml, which is 3.4 times higher compared to the middle age group. Similar trends were found in the concentration of IL-1. For the age group of 45–49, the concentration of IL-1 β was 3.38 pcg/ml, and in the age group of 80 years and older, levels of this cytokine increased almost 5 times. It was found that with age-related there is a gradual decrease in the level of IL-2, and a gradual increase of IFN-γ. The decrease in IL-2 is due to the typical aging decrease in the amount of T-lymphocytes.

**Conclusion:**

Thus, our results indicate that there are significant deviations of immune parameters, particularly in cytokine concentrations, in older adults compared to middle aged adults.

